# Synthetic fiber from a teddy bear causing keratitis and conjunctival granuloma: case report

**DOI:** 10.1186/1471-2415-11-17

**Published:** 2011-06-20

**Authors:** Mohammed K Farooq, Jan U Prause, Steffen Heegaard

**Affiliations:** 1 Department of Ophthalmology, Glostrup Hospital, University of Copenhagen, Denmark; 2 Eye Pathology Section, University of Copenhagen, Frederik V's Vej 11, DK-2100 Copenhagen, Denmark

## Abstract

**Background:**

To report a case of keratitis and a case of conjunctivitis caused by synthetic fibers from toy teddy bears.

**Case presentation:**

Case stories with histopathological analysis. 1) A two-year-old girl developed a severe case of keratitis and corneal ulceration. The initial treatment with various antibiotics gave no improvement and eventually the patient developed spontaneous perforation of the cornea. The corneal swabs contained no bacteria or fungi. Corneal grafting was performed and the corneal button was sent for histopathological examination. 2) A five-year-old girl presented with ocular irritation in her left eye. Examination revealed a conjunctival granuloma in the inferior fornix. The lesion was excised and histopathologically examined.

**Results:**

Microscopy revealed synthetic fibers embedded in the cornea and in the conjunctival granuloma. The diagnosis was confirmed by demonstration of marked birefringence of the synthetic fibers. Microscopical examination of synthetic fibers from two different types of fur (whiskers and face hairs) from the two-year-old girl's teddy bear was performed. Hairs from the face of the teddy bear were morphologically and microscopically identical with the fibers causing the severe corneal ulceration in the two-year-old girl.

**Conclusions:**

Doctors should especially in small children be aware of the risk of ocular consequences of close exposure of synthetic fibers from stuffed toy animals. Corneal ulceration, clinically presenting as corneal infection with negative culturing and staining, should lead to a different clinical strategy and treatment. The treatment of conjunctival synthetic fiber granuloma is excision and antibiotic eye drops.

## Background

A close exposure of the eyes of stuffed toy animals and blankets made of synthetic material may cause penetration of the conjunctiva or the cornea by synthetic fibers [1,2]. Usually, foreign bodies are removed from the ocular surface by the eye protective mechanisms as blinking and tearing. However, foreign bodies may be retained and encapsulated by the mucous and initiate a local inflammatory response [3-5]. This may cause granuloma formation in the conjunctiva and in case of retained synthetic fibers, the lesion is known as "teddy bear granuloma", first described by Weinberg et al in 1984 [1]. Sharp lesions from animal hairs, like hairs from tarantula and caterpillar may also penetrate the corneal surface and give rise to serious intraocular conditions [6-9].

Fifteen cases of conjunctival teddy bear granuloma have been published [2], but so far no case of keratitis caused by synthetic fibers from a toy teddy bear has been reported.

We report a unique case of keratitis and a case of conjunctival granuloma caused by synthetic fibers from toy teddy bears.

## Case presentation

### Case 1

A two-year-old girl was referred by her general practitioner. Three weeks previously the patient presented with ocular irritation and corneal opacities of her left eye. Topical treatment with ciprofloxacin and chloramphenicol had no improvement of the condition. According to the mother there had been no trauma or foreign body complaints in the patient. The girl was born with oesophageal atresia, and had mild asthma. Apart from this she was in a healthy medical condition.

**Figure 1:**
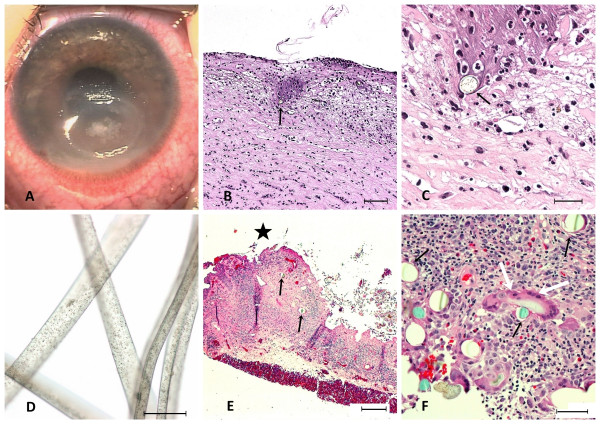
**Corneal (*A-D*) and conjunctival granuloma (*E-F*) caused by synthetic fibers from a toy teddy bear**. *A*: A 4 × 5 mm oval corneal ulceration due to synthetic fibers in the 2-year-old girl (*case 1*). *B-C*: Haematoxylin-eosin (HE) staining of the inflamed corneal biopsy. The stroma contains multiple histiocytes and neutrophils and is embedded with bluish brown synthetic fibers (black arrows) exhibiting central black granular spots. *Bars: 150 μm (B), 50 μm (C)*. *D*: Microscopy of synthetic fibers from the face of the 2-year-old girl's teddy bear causing the corneal ulceration. *Bar: 50 μm*. *E-F*: HE stained conjunctival biopsy from the 5-year-old girl (*case 2*) showing a normal differentiated epithelium (star = conjunctival surface). The connective tissue is infiltrated with inflammatory cells mostly eosinophils, and synthetic fibers (black arrows) surrounded by foreign body giant cell (white arrows). *Bars: 200 μm (E), 50 μm (F)*.

Examination in general anaesthesia revealed a 4 × 5 mm oval ulceration infero-centrally of the left cornea with purulent exudate, stromal oedema and a couple of large "mutton fat" precipitates (Figure 1A). No foreign bodies were detected. In addition a severe iritis with synechiae and a hypopyon less than one mm was found. Conjunctival and corneal swabs were sent for bacterial and fungal staining and culture. Due to a clinically suspected infection, topical treatment was changed to oxytetracyclin-polymyxin-B ointment, ciprofloxacin and chloramphenicol eye drops six times daily and topical atropine once daily.

For the next two weeks there was a remarkable improvement of the corneal ulceration. However, suddenly a relapse with a new hypopyon and worsening of the corneal ulceration occurred. The treatment was supplemented with fortified gentamicin and fortified cefuroxin eye drops every hour. One week later the results of the staining and the cultures were proved negative for bacteria and fungi. The condition worsened and a spontaneous perforation occurred centrally in the ulceration. Furthermore, a new but smaller ulceration was observed more laterally. Corneal transplantation was performed and the corneal button was sent for microscopical examination.

In addition, microscopical examination was performed on synthetic fibers from two different types of fur (whiskers and hairs from the face) from the child's toy teddy bear.

### Case 2

A five-year-old girl was admitted to the eye department because of irritation and foreign body sensation in her left eye for two days. She was otherwise in a good general health.

Slit lamp examination showed a 7 × 5 mm granuloma in the left inferior conjunctival fornix embedded with hairs and synthetic fibers. The lesion prolapsed easily with gentle pressure on the left lower lid. The examination was otherwise normal.

The lesion was excised under general anaesthesia and sent for microscopical examination. Postoperatively, the eye was treated with chloramphenicol three times daily for five days. The wound healed within two weeks and the postoperative course was uneventful.

### Histopathology

The specimens were fixed in 4% buffered formaldehyde and embedded in paraffin. Sections were cut at 4 μm and mounted on glass slides.

Deparaffinized sections were stained with haematoxylin-eosin (HE), periodic acid-Schiff (PAS), colloidal iron (CI), Alcian blue at 1.0 and 0.2 mol MgCl_2 _and Gordon & Sweets' method for reticular fibers.

## Results

### Case 1

The light microscopy showed a heavily inflamed and ulcerated cornea (Figure 1B). Bowman's layer was missing and the ulceration occupied most of the stroma reaching Descement's membrane. The stroma contained a granulomatous lesion with multiple histiocytes and neutrophils. Numerous round to oval shaped blue and brown fibers with black granular spots were seen centrally in the lesion (Figure 1C). The diameter of the fibers ranged from 20-30 μm and showed strong birefringence in polarized light (Figure 1D).

To verify whether the synthetic fibers belonged to a toy teddy bear, we performed microscopical examination of two different types of synthetic fibers (whiskers and face hair) from the girl's toy teddy bear. Hairs from the face of the teddy bear were morphologically and microscopically identical with the fibers causing the severe corneal ulceration in the two-year-old girl.

Investigation of the teddy bear fur demonstrated that the fiber was made of 100% polyethylene (Dacron, Terylene). The azo-dye (Faron Dark Blue, Clariant), applied to the fibers is not restricted by EU and is not known to cause inflammation.

### Case 2

Microscopical examination of the conjunctiva revealed a normal differentiated conjunctival epithelium with goblet cells (Figure 1E). The subepithelial stroma contained a granuloma with inflammatory cells, mostly eosinophils, histiocytes and foreign body giant cells (Figure 1F). Synthetic fibers, identified by their strong birefringence in polarized light were found within the lesion. The diameter of the fibers ranged from 20-30 μm. We could not identify the chemical composition of these fibers.

## Conclusions

Increased tear flow, redness and foreign body sensation are the initial symptoms associated with foreign body in the eye, followed by pain, photophobia and ciliary injection. Signs of possible deeper ocular penetration include decreased vision and clouding of the cornea [10].

The majority of patients with conjunctival granuloma (caused by synthetic foreign bodies) were referred to an eye department after the granuloma was visible [2]. This may in children take weeks since children may neglect the symptoms until the granuloma has developed, or because the symptoms communicated to the parents are misunderstood [2,11]. However, in the present case of conjunctival granuloma signs were seen early and the child was referred after two days.

Even though the patient with the corneal lesion was examined with slit lamp, the examiner was unable to identify the corneal foreign body. A main reason might be that the synthetic fibers were bluish brown similar to the colour of the hypopyon and the iris. However, a slit lamp examination combined with negative bacterial and fungal staining and culturing should lead to a diagnosis of foreign body granuloma.

Cases with tarantula hairs and caterpillar setae penetrating the cornea and giving rise to serious conditions like chronic kerato-conjunctivitis, intracorneal hairs with granuloma formation and chronic iritis are well documented [6-9]. However, so far no similar consequences of synthetic fibers from a teddy bear's hair have been reported. The lesions are considered partly due to infiltration and to the inflammatory/toxic reactions, in the same way as hairs from the tarantula back are supposed to irritate the skin [12]. Whether the same mechanisms are responsible for the severe case of keratitis in our patient is unclear. However, the synthetic fibers in case one were made of polyethylene. When used in joint arthroplasty, polyethylene may cause an osteolytic reaction when small particles are engulfed by macrophages in granulomatous reactions [13]. A similar reaction could be the explanation in our patient. The azo dye applied in the fibers may also have had an influence, however, it has not been documented to have any adverse effect.

In a recent review of the literature, Schmack et al demonstrated the histopathological and ultrastructural features of conjunctival granuloma caused by synthetic fibers [2]. The diagnosis is confirmed by the microscopic features of the conjunctival granuloma showing granulomatous inflammatory tissue with lymphocytes, plasma cells, eosinophils and usually foreign-body giant cells surrounding the synthetic fibers. The simplest method to confirm the diagnosis is excision of the conjunctival granuloma and microscopical examination demonstrating marked birefringence of the synthetic fibers when examined in polarized light [14,15]. The synthetic fibers are fairly uniform in diameter and generally round to oval in cross sections. The diameter of the synthetic fibers in the cases published by Schmack et al and Weinberg et al ranged from 17-29 μm and 21-27 μm like in our case [1,2]. The localization of the granuloma in the previously published 15 cases was unilateral and mainly in the inferior fornix, except in one case in the superior fornix [2].

The ocular surface epithelium not only acts as a physical and mechanical barrier against harmful substances, but also participates actively during allergic inflammatory processes [16]. Patients with asthma might react with a profound ocular hypersensitivity [17]. Thus the formation of a conjunctival granuloma in case two, the patient with known asthma could have been potentiated by local ocular allergic reactions.

Surgical removal of the conjunctival granuloma and postoperative treatment with antibiotics is recommended and has shown to be successful [14,18]. The crucial element in the treatment of keratitis is the identification of the cause of the keratitis. In young children rare causes such as synthetic fibers should always be kept in mind, especially in children who are attached to their toy teddy bears. Identification and removal of the corneal and conjunctival foreign body granuloma followed by antibiotic administration are the treatment of choice.

## Competing interests

The authors declare that they have no competing interests; there was no research funding and no proprietary interests in the materials described.

## Authors' contributions

MKF performed the literature review and drafted the manuscript. SH and JUP performed the microscopical examination of the cornea and the conjunctival granuloma and assisted in the final preparation and submission of the manuscript. All authors read and approved the final manuscript.

## Pre-publication history

The pre-publication history for this paper can be accessed here:

http://www.biomedcentral.com/1471-2415/11/17/prepub
